# Baseline Frequency of Chromosomal Aberrations in Control Donors: A Significant Value for Population Monitoring During the Development of the Nuclear Energy Industry—Polish Dataset

**DOI:** 10.3390/toxics14080659

**Published:** 2026-07-27

**Authors:** Sylwester Sommer, Iwona Buraczewska, Anna Korzeniowska-Sobczuk, Katarzyna Sikorska

**Affiliations:** Institute of Nuclear Chemistry and Technology, 03-195 Warsaw, Poland; i.buraczewska@ichtj.waw.pl (I.B.); a.sobczuk@ichtj.waw.pl (A.K.-S.); k.sikorska@ichtj.waw.pl (K.S.)

**Keywords:** chromosomal and chromatid type aberration nomenclature, ionizing radiation exposure, background level of chromosomal aberrations, nuclear industry, genotoxic effect of ionizing radiation

## Abstract

Due to the advanced plans for the development of nuclear power plants in Poland, those who are to live around nuclear facilities may have unjustified doubts about their own radiation safety. Moreover, when future power plant workers will be potentially exposed to low, cumulative doses of ionizing radiation, it seems important to develop cytogenetic methods for showing the actual genotoxic effects of environmental or occupational nuclear influences. The method of analyzing the frequency of chromosomal type aberrations in peripheral blood lymphocytes remains one of the most significant possibilities in achieving this goal. Three techniques are used to monitor aberration frequency, i.e., most commonly, Giemsa staining (FPG), FISH, or M-FISH. The types of aberrations determined by Giemsa-staining preparation will be described in detail, and issues with the nomenclature of these aberrations will be discussed. To effectively explore small changes in aberration yields, studies of aberration rates in non-exposed populations are needed. Such a dataset has been generated at the Institute of Nuclear Chemistry and Technology (INCT), Warsaw, Poland. This database would allow for the assessment of the genotoxic impact of planned nuclear facilities, the impact of radon in areas within its increased concentration, or the impact of such a working environment on, for example, medical workers working with radiation.

## 1. Introduction

The development of the nuclear power industry in Poland will potentially expose large groups of people to low doses of ionizing radiation, including the exposure of nuclear facility workers to low doses associated with routine work and the risk of (higher) emergency doses; as well as exposure of people living near the power plant to low (negligible) doses associated with the plant’s operation. Because ionizing radiation is a genotoxic agent and is therefore considered a risk, it is crucial to develop methods that can estimate the genotoxic effects of radiation on the human population. Analysis of the frequency of aberrations in peripheral blood lymphocytes remains the method of choice, as it is the best-described, validated, and most sensitive method currently available [1,2]. The method is inexpensive, widely used, and considered the gold standard by the International Atomic Energy Agency [1]. Other cytogenetic techniques, such as various types of micronucleus assays, the comet assay, sister chromatid exchange analysis, and others can also be used to determine genotoxic effects; however, these will not be discussed in this article [1,2].

**Figure 1:**
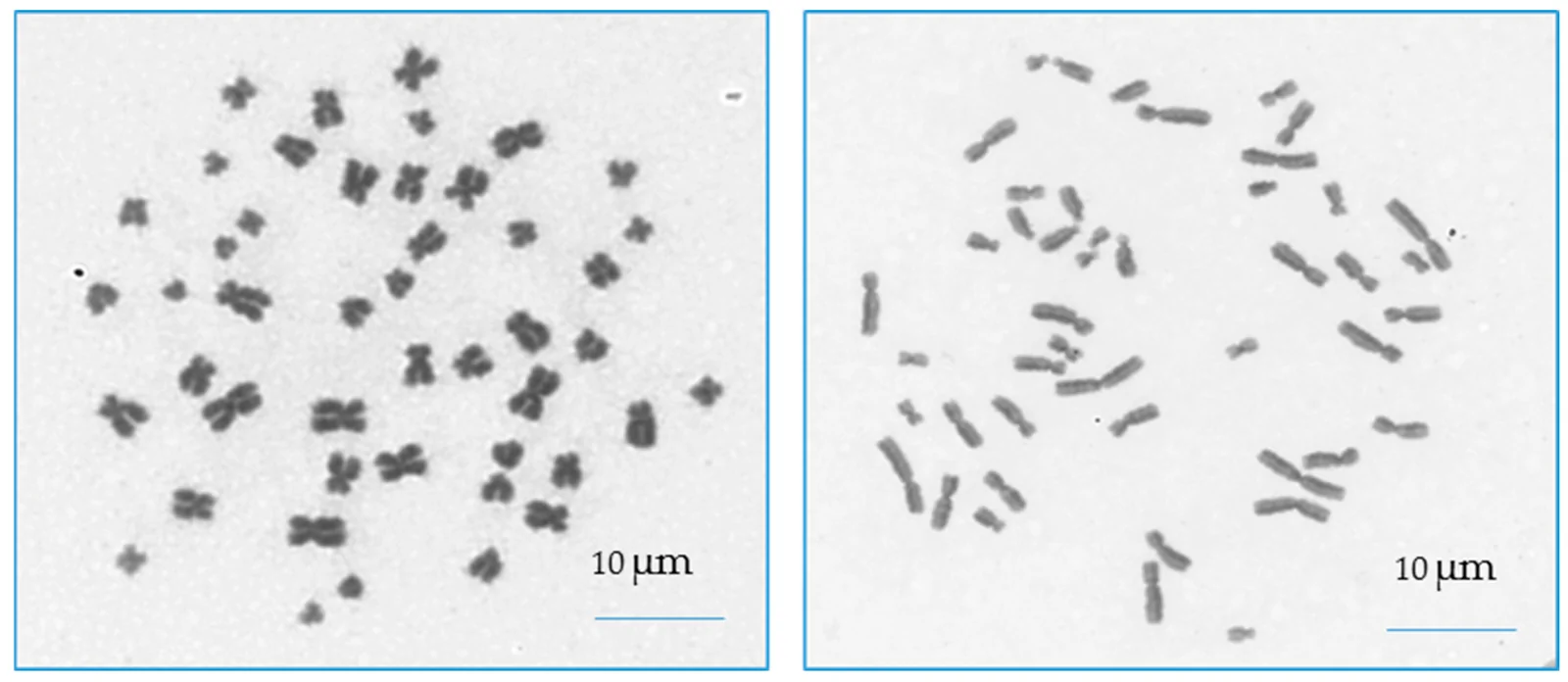
Undamaged human lymphocyte in mitosis—FPG staining [30]. Chromosomes appear without any morphological changes. Left panel shows uniformly stained chromosomes in first mitosis after the stimulation of cells for division. Both chromatids are uniformly stained. Right panel shows chromosomes in second mitosis following division stimulation—chromatids are differentially stained (harlequin chromosomes).

**Figure 2:**
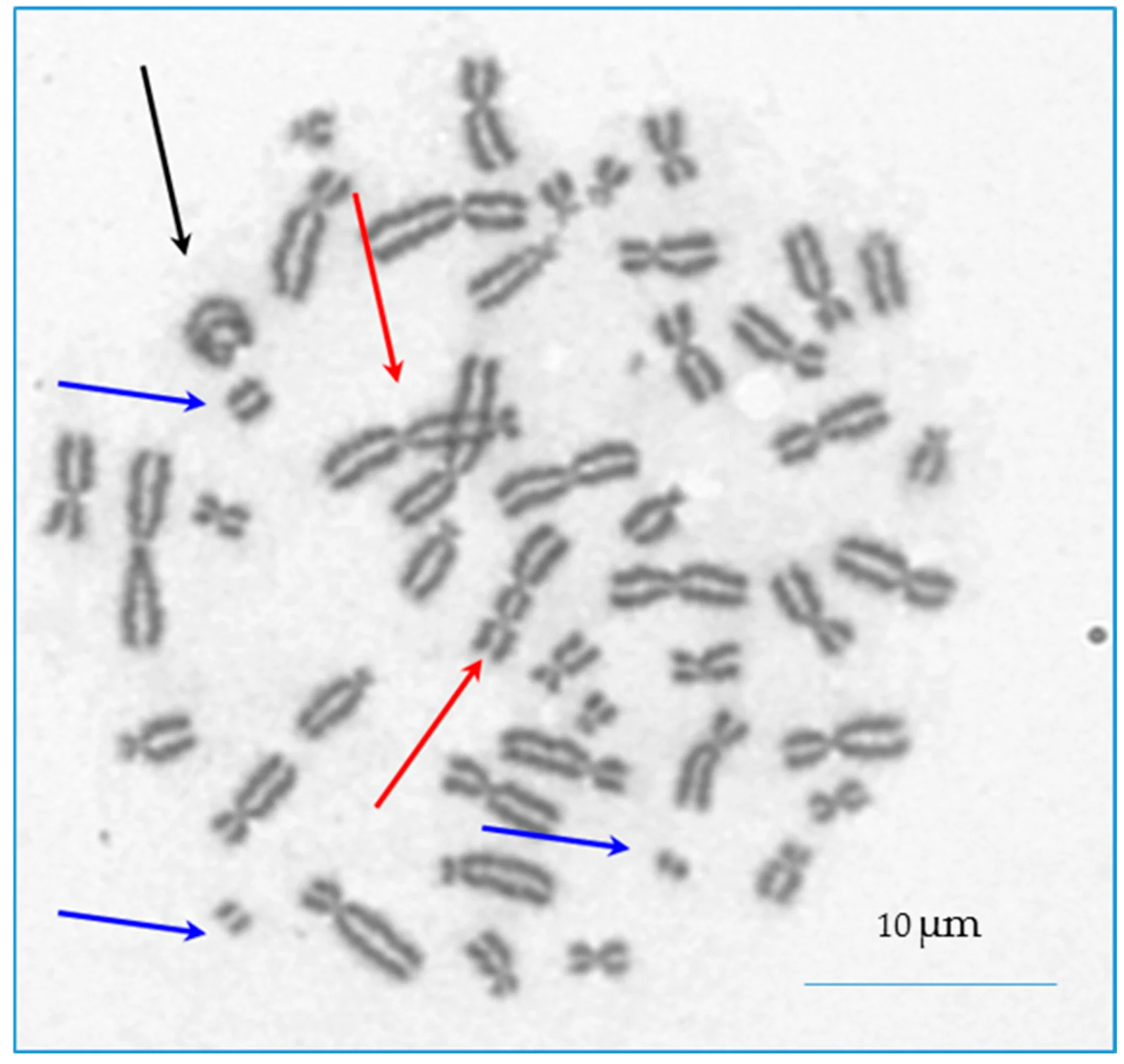
Different chromosomal aberrations in human lymphocytes irradiated with ionizing radiation (gamma Co-60, Isledovatel, dose rate 0.66 Gy/min) [30]. Different types of aberrations are indicated with arrows: red arrows—dicentric chromosomes; black arrow—centric ring. Acentric fragments which, in this situation, are parts of other aberrations (accompanying dicentric chromosomes and the centric ring), are indicated with blue arrows.

**Figure 3:**
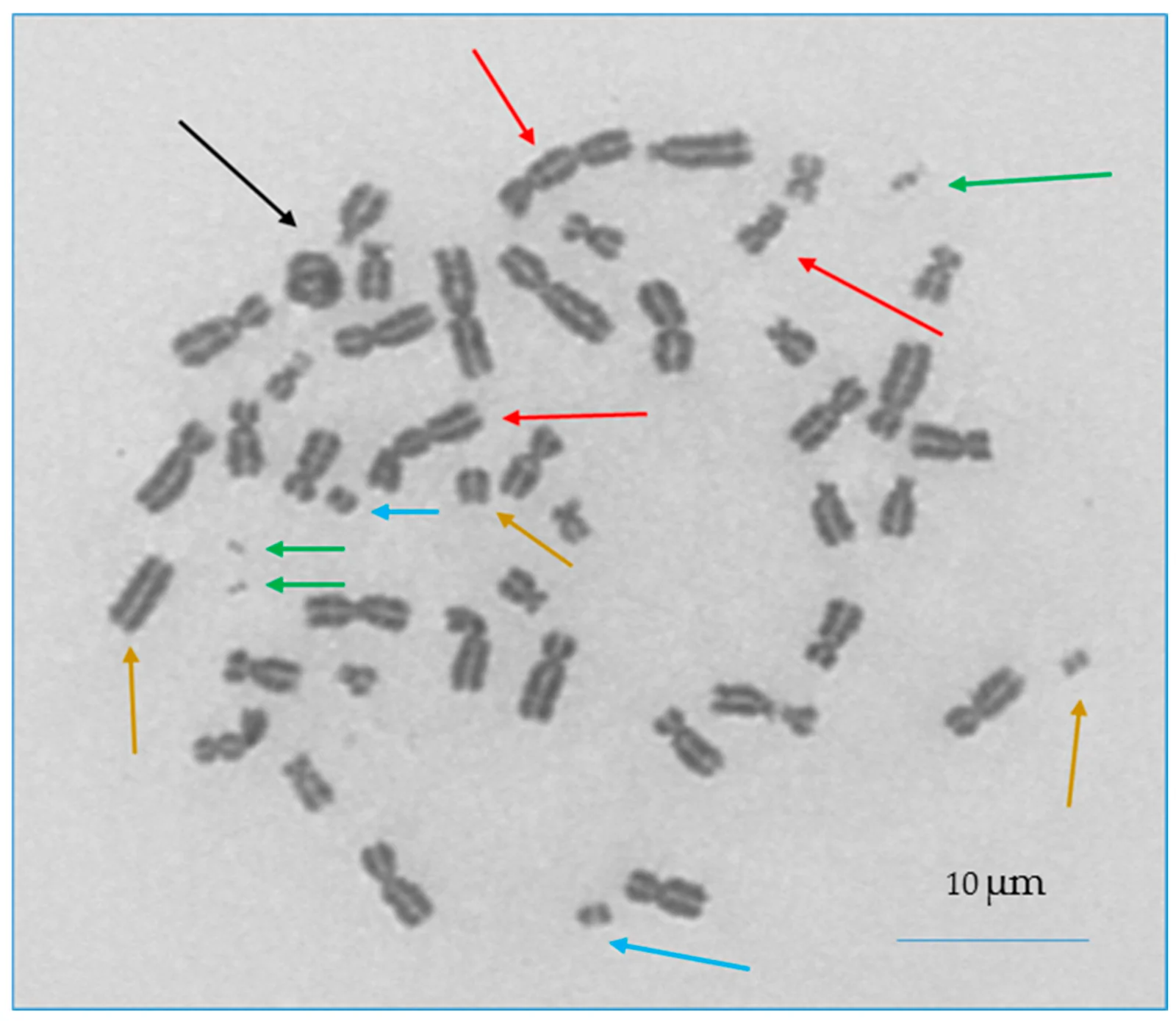
Different chromosomal aberrations in human lymphocytes irradiated with ionizing radiation (X-rays, YXLON portable X-ray flaw detector, dose rate 1.1 Gy/min) [30]. Different types of aberrations are indicated with arrows: red arrows—dicentric chromosomes; black arrow—centric ring. Eight acentric fragments of different shapes and lengths, are indicated with arrows in three colors: green arrows—acentrics resemble double minutes; blue arrows—acentrics resemble acentric rings; brown arrows—acentrics with a pronounced length.

**Figure 4:**
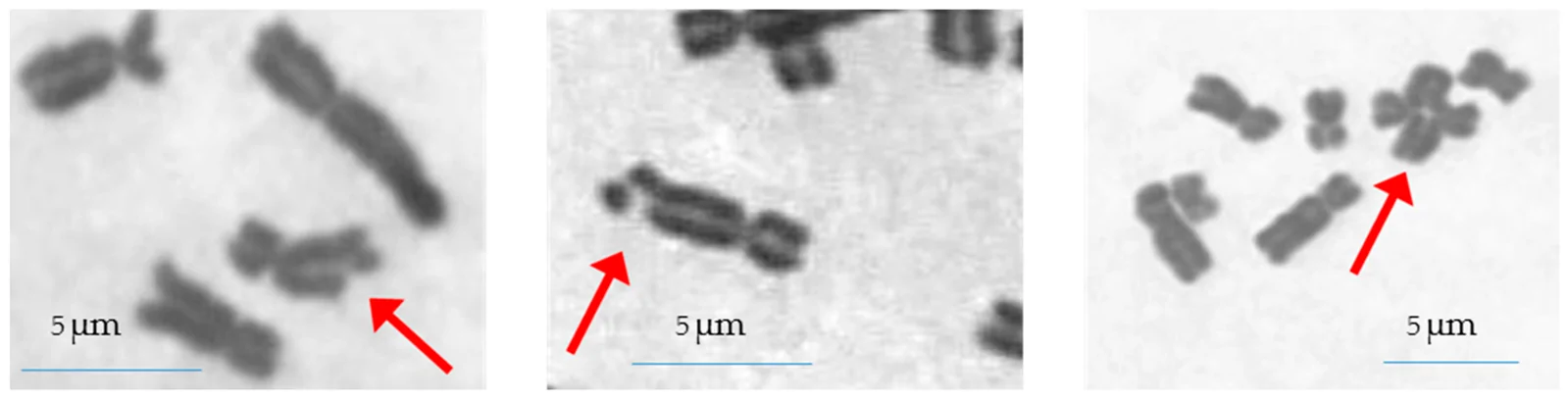
Chromatid aberrations in human lymphocytes, non-irradiated sample [30]. The two photos on the left show chromatid breaks. The photo on the right shows chromatid exchange (symmetrical complete chromatid exchange, quadriradial) [1,30].

**Figure 5:**
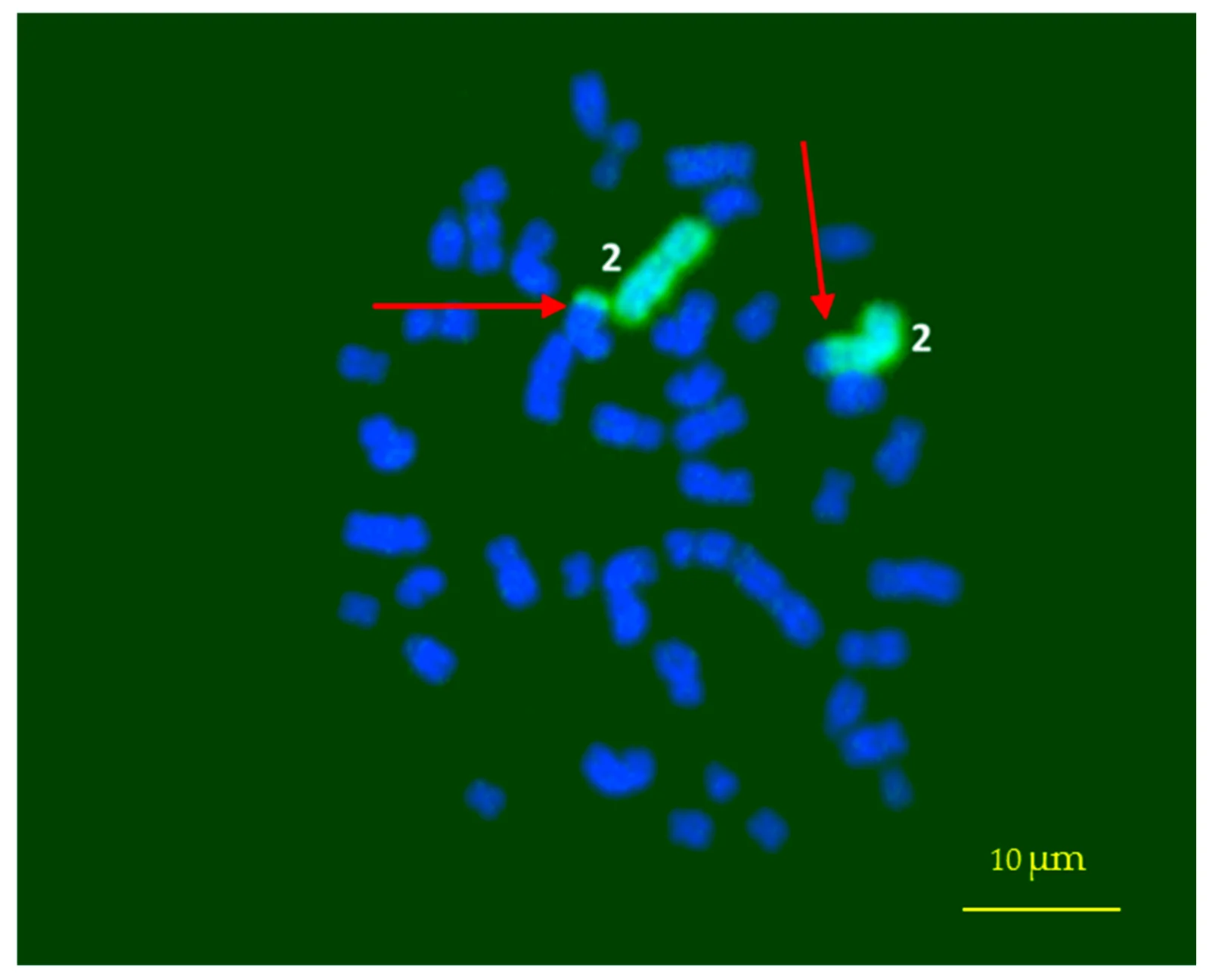
Peripheral blood human lymphocyte in mitosis, irradiated with 2 Gy of X-rays (YXLON portable X-ray flaw detector, dose rate 1.1 Gy/min), DAPI stained, with one pair of chromosomes painted green using FISH technique [30]. Reciprocal translocation between FISH-painted chromosome 2 and the DAPI-stained chromosome (red arrows) is visible.

**Figure 6:**
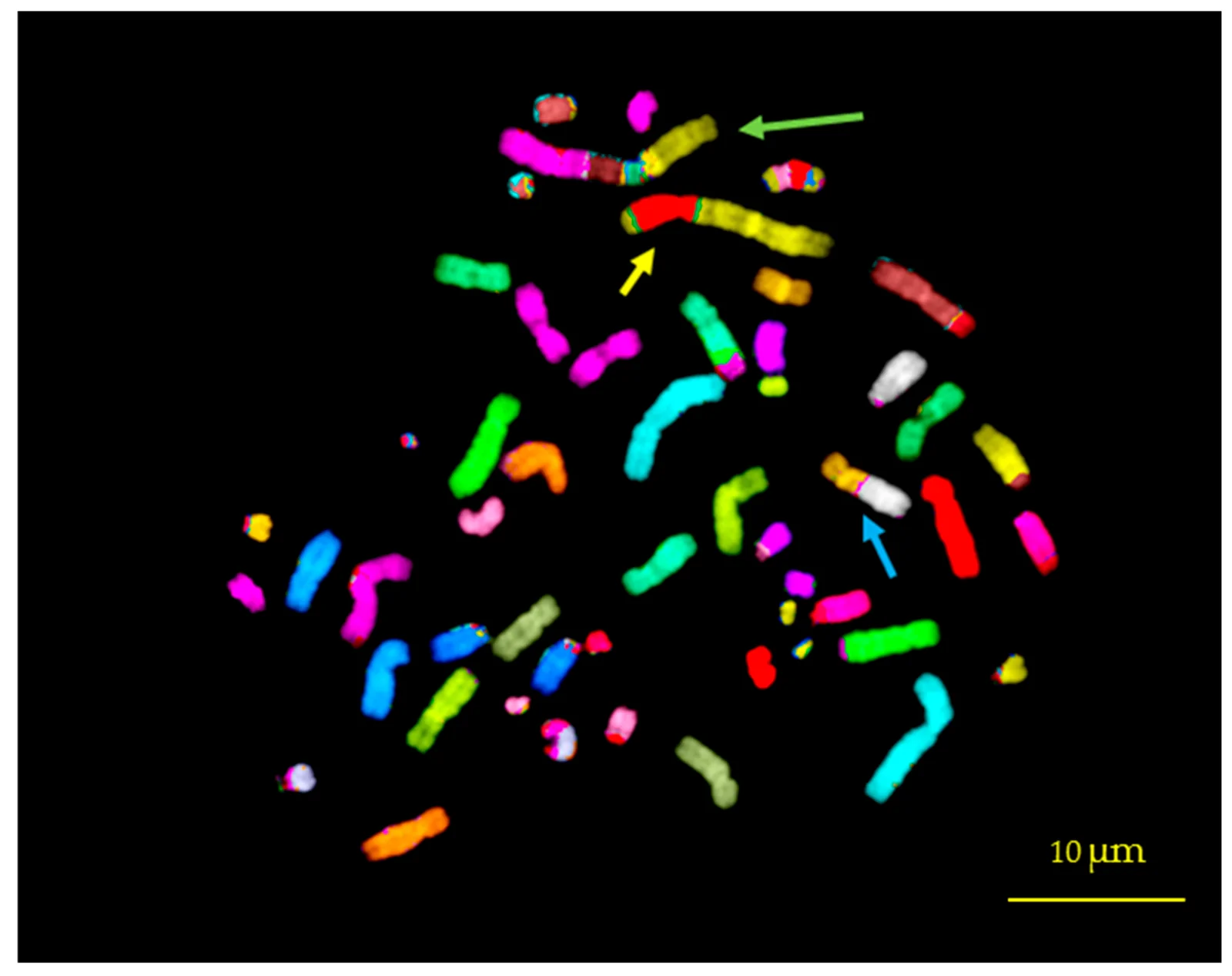
Human peripheral blood lymphocyte in mitosis, irradiated with 2 Gy of C-ions (carbon ions, at GSI Helmholtzzentrum für Schwerionen-forschung GmbH Darmstad, Germany), stained using the M-FISH method [30]. Numerous chromosomal aberrations are visible, including a few complex types. There are so many aberrations that only three are pointed out with arrows. These indicated aberrations would not be entirely visible if stained with Giemsa (FPG). The green arrow indicates a tricentric chromosome consisting of genetic material from four different chromosomes. This chromosome is a part of larger complex aberrations. The yellow arrow points to a dicentric chromosome with an insertion, which is also part of a more complicated complex aberration. The blue arrow points to a translocation between two chromosomes.

When exposed to low doses of radiation, three methods of aberration analysis are possible: standard FPG staining (Fluorescent Plus Giemsa) (Figure 1, Figure 2, Figure 3 and Figure 4), the expensive translocation analysis using chromosome painting (usually two or three pairs of homologous chromosomes) (Figure 5), and the most expensive M-FISH technique, painting all the chromosomes (Figure 6). All three methods have their advantages and disadvantages, which will be described in more detail later in this article. The FPG staining method is the simplest and least costly [1]. Using this technique, a variety of different aberrations can be identified, including dicentric chromosomes, excess acentric fragments, and certain chromatid type aberrations; however, the frequency of translocations cannot be analyzed using this method [1]. There are some discrepancies in the nomenclature of aberrations in mitoses stained using the FPG method, i.e., the classification of acentric fragments remains particularly unclear. Chromosome painting of two to several pairs of homologous chromosomes (FISH), in addition to all types of aberrations analyzable via the FPG method, allows for the analysis of translocations and complex aberrations within the painted fragment of the genome [1,2]. This method is more expensive than FPG staining, as it requires the usage of costly fluorescent probes and the use of a fluorescence microscope. The M-FISH technique, being the most precise method, comprises the labeling of all chromosomes with DNA probes and the analysis of the frequency of translocations and complex aberrations across the whole genome.

All the aforementioned methods compare the observed aberration frequencies with those found in the background cells. While at higher radiation doses, the aberration frequencies exceed the background values many times over, at low doses, they remain close to the control levels. However, even then, through epidemiological studies, it is possible to link these slightly elevated frequencies with an increased risk of developing cancerous diseases, cardiovascular diseases, and other health issues [2,3,4,5,6,7,8,9,10,11,12]. Such studies have been and continue to be conducted in many countries in the context of radiation exposure related to the nuclear power industry, occupational exposure, or living in areas with elevated radon concentrations [2].

To determine the genotoxic effect of low radiation doses, it is essential to compare the study group with a background level of aberrations database. Such a database should be sufficiently large and account for a range of factors, such as the donor’s age, gender, smoking habits, geographic and ethnic diversity, and other variables. These databases were created by laboratories specializing in the genotoxic effects of radiation; for instance, they have been developed through international scientific projects. More recently, large sets of data from individual countries are being published. It appears that in Poland, a valuable database of aberrations in control donors was established only once, 25 years ago, in Silesia. Given, on the one hand, the development of the nuclear power industry and methods involving ionizing radiation in industry and medicine, and on the other, the possibility of utilizing motorized microscopes coupled with high-resolution CCD cameras for aberration frequency analysis—which offers superior analytical capabilities—it would be highly desirable for such a database to be established in Poland today. At the Institute of Nuclear Chemistry and Technology (INCT) in Warsaw, we are currently working on creating this type of database. To date, approximately 13,000 cells, sourced from 13 donors with no history of exposure to genotoxic factors and exclusively from Warsaw and the surrounding areas, have been analyzed using the FPG staining technique. The database will be expanded with additional participants as resources permit.

Cytogenetic techniques, which allow for the monitoring of genotoxic effects, can be utilized to analyze the impact of not only low radiation doses originating from nuclear power, but also:

- the effects of radon (as well as radium or uranium) in areas with elevated concentrations [13,14,15];
- the influence of the occupational environment on, for example, medical personnel working with radiation [2,16,17,18,19], or workers in the tobacco industry [20,21];
- the impact of environmental pollution on the general population [22,23,24,25,26,27].

It is important to remember that an increased frequency of aberrations is a characteristic of both cancer cells and “healthy” cells in individuals with cancer, and that one-third of us will develop cancer at some point in our lives [12].

The primary aim of this paper is to underline the importance of having a reliable method to evaluate the cytogenetic burden of people working in or living close to nuclear power installations. Three cytogenetic techniques are described, with an emphasis on FPG staining. Problems with aberration nomenclature in Giemsa-stained slides are discussed, particularly regarding excess acentric fragments. To support the claim that the analysis of aberrations in Giemsa-stained slides remains the method of choice, baseline frequency datasets of chromosomal aberrations in control donors are presented from more than 15 countries. Finally, a preliminary dataset of baseline aberrations in the Polish population is presented.

## 2. Methods for Analyzing Aberration Frequency in Population Monitoring and Types of Aberrations

### 2.1. Chromosome Staining Using Giemsa Staining (FPG)

The standard method for staining preparations to assess aberration frequency is Giemsa staining. This method is extensively described and standardized [1,28]. Additionally, a thymidine analog, which incorporates into the replicating DNA strand, is added to the lymphocyte cultures, enabling FPG staining [1,29]. FPG staining allows for the differentiation between the first mitotic divisions following lymphocyte stimulation and subsequent divisions (Figure 1).

Giemsa staining (or FPG) is the most frequently chosen chromosome staining method because it is simple and inexpensive. In the case of analyzing the background level of the aberrations and studying the genotoxic impact of the environment or the workplace, only a few classes of aberrations are analyzed, and these are often combined into groups. Typically, the frequency of unstable chromosomal aberrations, such as dicentric chromosomes, centric rings, and excess acentric fragments, is analyzed (aberrations visible in Figure 2 and Figure 3) [1,31,32,33,34,35]. Additionally, some researchers count the frequencies of translocations when they are visible (however, most of them remain invisible with Giemsa staining, so counting them is dubious) [33,35]. Besides chromosomal aberrations, chromatid aberrations, such as chromatid breaks or chromatid exchanges, are analyzed (Figure 4). Not all types of aberrations are taken into account, as some are complex, difficult, or impossible to identify and/or are very rarely encountered [1]. For statistical analysis, all types of aberrations may be combined into a single group, namely total aberrations, or chromosomal and chromatid aberrations may be analyzed separately.

A detailed description of all types of aberrations is contained in the following publications: Chromosome Aberrations Induced by Ionizing Radiation [36], Classification and Relationships of Induced Chromosomal Structural Changes [31], Cytogenetic Dosimetry: Applications in Preparedness for and Response to Radiation Emergencies, EPR-Biodosimetry (IAEA 2011) [1], International System for Human Cytogenetic Nomenclature (issued periodically since 1985) [37], and Mechanisms of Clastogen-Induced Chromosomal Aberrations: A Critical Review and Description of a Model Based on Failures of Tethering of DNA Strand Ends to Strand-Breaking Enzymes [32]. However, aberration classification systems are not entirely consistent. This is partly due to the fact that chromosomal aberrations can be examined using various types of preparations, including staining with Giemsa dye (FPG), Giemsa-staining with various types of banding (G-banding, R-banding, Q-banding), fluorescence staining of chromosomes FISH (or M-FISH), or through mixed techniques, such as DAPI chromosome staining with fluorescent staining of telomeres or/and centromeres. Additionally, different aberrations are observed, depending on the stage of the cell cycle at which the genetic material of the cells was damaged; damage in the G0 or G1 phase results primarily in chromosomal aberrations, whereas damage in the G2 phase leads mainly to chromatid aberrations.

A large number of studies containing data on control aberration frequencies comprise those monitoring the impact of the occupational environment (e.g., medicine) on aberration frequency, and they are conducted by scientists with a background in clinical cytogenetics. Despite the fact that the technique used includes the analysis of aberrations in peripheral blood lymphocyte preparations uniformly stained with Giemsa (FPG), the aberration nomenclature aligns more closely with banded preparations (G-banding) and appears to be based on the International System for Human Cytogenetic Nomenclature (ISCN) publications [37]. This type of analysis is more suitable for assessing genetic disorders and translocation levels rather than environmental impact.

Meanwhile, the control group for cytogenetic monitoring studies should consist of individuals who have not been subjected to overt exposure to genotoxic chemical or physical agents or repeated medical procedures involving exposure to ionizing radiation and who are genetically healthy (without genetic syndromes or diagnosed neoplastic disease). From such individuals, we examine peripheral blood lymphocytes, which are mostly in the G0 phase of the cell cycle. We therefore expect that most of the aberrations found will be unstable (asymmetric) chromosomal aberrations, such as dicentric chromosomes, centric rings, and excess acentric fragments, while chromatid-type aberrations will only comprise a minor addition. Additionally, we are aware that stable aberrations such as translocations are largely invisible in Giemsa-stained preparations without banding.

Based on the above considerations and our extensive experience (approximately 150,000 analyzed control peripheral blood lymphocytes stained with FPG), we present a list of the types of aberrations that are visible in the Giemsa-stained preparation.

Chromosomal type aberrations:

- Dicentric chromosomes

Figure 2 and Figure 3 (red arrows). Usually accompanied by acentric fragments (blue arrows). An asymmetrical exchange between two chromosomes (an unstable chromosomal aberration) [1,31]. The most frequently analyzed type of aberration, selectively induced by ionizing radiation and used for dose reconstruction in biological dosimetry.

- Centric rings

Figure 2 and Figure 3 (black arrows). Usually accompanied by an acentric fragment (blue arrows). An asymmetrical exchange between the arms of the same chromosome (an unstable chromosomal aberration) [1,31]. In human peripheral blood lymphocytes, they are significantly rarer than are dicentric chromosomes. Centric rings are often combined into a single group with dicentrics, for example, in biological dosimetry [1].

- Acentrics (fragments)

Figure 3 (blue, brown, and green arrows): A chromosomal aberration or the visible result of various types of aberrations—a fragment without a centromere. They appear under various names, most commonly acentric fragments [2,22,37], excess acentrics, excess acentric fragments [23], excess fragments [38], breaks [38,39], chromosome breaks [2,40] (only if the resulting acentric fragment and the abnormal chromosome are part of a single event [37]), chromosome-type breaks [38], chromosome fragments [38], isochromatid deletions [1,41], isochromatid breaks [24,35,37], or isolocus breaks [37]. In irradiated lymphocytes, acentric fragments reflect various types of aberrations (interstitial deletions [1,42], i.e., acentric rings [2,34] or minutes [23,37,39], including single minutes [37] or double minutes [37]; terminal deletions; incomplete chromosomal exchanges; and isochromatid deletions). Since aberrations in lymphocytes originate in the G0 phase of the cell cycle, a high frequency of chromatid aberrations, including isochromatid deletions, is not to be expected, as their formation is primarily associated with the G2 phase. Given that the observable morphological change—an acentric—is identical to that seen in chromosomal aberrations, it can be assumed that the majority of such changes are derivatives of either interstitial deletions or terminal deletions; therefore, the term “isochromatid deletion” should be avoided in this context.

As studies using telomeric PNA probes in irradiated lymphocytes indicate, at their ends, acentrics may possess two sets of telomeres (in which case, they are likely the result of an event involving at least two breaks on two chromosomes), one pair of telomeres (resulting from a terminal deletion), or no telomeres at all (in which case, they are interstitial deletions, statistically shorter than acentrics with one or two telomeres, and identified as acentric rings if a lumen is visible, or as minutes if their length is less than the width of the chromatid) [1,37,43,44,45]. These results are the ultimate proof that acentrics are a group of aberrations with a different origin. For obvious reasons (the exceptionally low frequency of acentrics), studies using telomeric PNA probes have not been conducted on control preparations. Consequently, the number of telomeres in acentrics on control slides remains a matter of speculation.

The criterion for identifying minutes (a length smaller than the width of the chromatid) is sufficiently vague, and acentric fragments in the background so infrequent, that distinguishing these types of changes may be impractical. Minutes are often associated with extra DNA material amplified in certain cancer cells [46]; however, gene amplification is unlikely to occur in the lymphocytes of healthy donors. Acentrics that resemble minutes are encountered in lymphocytes within so-called rogue cells—cells containing multiple aberrations that occur rarely in control preparations. In a rogue cell, we often observe several dicentric chromosomes and numerous acentrics, which are very short and appear as minutes. The origin of rogue cells is not fully understood [1,47]. Typically, aberrations found in rogue cells are excluded from the calculated aberration frequencies unless there is an indication that the individual was exposed to high-LET radiation, which, again, is not the case for control subjects [1].

In summary, it is necessary to standardize the nomenclature of morphological changes that appear as acentrics (acentric fragments). The preceding paragraphs describe how such morphological changes can result from various types of aberrations, including interstitial deletions (acentric rings or minutes, double minutes), terminal deletions, incomplete chromosomal exchanges, or isochromatid deletions (Figure 3). In the majority of cases involving Giemsa-stained preparations, it is impossible—and indeed unnecessary—to identify exactly which specific aberration an acentric fragment represents based solely on its appearance (Figure 3). For example, in Figure 3, there are eight acentric fragments with different appearances. Green arrows indicate acentrics resembling double minutes, blue arrows indicate acentrics resembling acentric rings, and brown arrows show acentrics with a pronounced length, especially with one being longer than half of all normal chromosomes. There are four additional aberrations in the mitosis; i.e., three dicentric chromosomes and one centric rings; each of these aberrations should be accompanied by acentric fragments. Thus, four remaining acentrics are in fact excess acentric fragments, but it is not possible to identify which is which. Their origins may be different. Therefore, acentric fragments should be treated as a collective category of aberrations. A review of the literature clearly shows that this set of aberrations is categorized and named differently across various publications. For readers not specialized in the mechanisms of aberration formation, this creates chaos and the impression that different studies are identifying entirely different types of aberrations—which is not strictly true. It appears that the most commonly used terms are acentrics, acentric fragments, or excess acentric fragments, and one of these names is consistently selected.

Chromatid type aberrations:

- Chromatid breaks

Figure 4 shows chromatid breaks [1,2,37,40], minutes [23,39], and chromatid deletion only in banded preparations, when there is an absence of a banded sequence on only one of the two chromatids [37,41,48]. These affect only one chromatid, and the unstained region (gap/break) should be wider than the width of the chromatid [1].

- Chromatid exchanges, Figure 4. Chromatid exchanges [1,2,37,49] or radial configurations [16]: triradials [1,17,18,31,37], tetraradials [17,18], quadriradials [31,35,37], complex [37] chromatid inversions (only in banded preparations [37]). Theoretically, there are several forms of these aberrations, and a detailed description is provided by Savage (1976) [31]. The most common form, often referred to as a tetraradials or quadriradials, is a symmetrical exchange corresponding to dicentric chromosomes (Figure 4) [1,41]. Much less frequently, triradials (three-arm configurations) are also observed [1]. If more than two chromosomes and/or more than two breaks are involved in the aberration, it is classified as a complex aberration [37].

The list of aberrations provided above is neither definitive nor exhaustive. More detailed descriptions of aberration classification systems can be found in the previously mentioned works by Evans and Savage [31,36]. Nevertheless, these five types of aberrations are the ones most frequently observed in the human lymphocytes of control donors.

In addition to chromosomal and chromatid aberrations, staining issues within chromatid regions—known as gaps, achromatic lesions, or chromatid gaps (if they affect only one chromatid)—are also observed [1,37]. The exact nature of these changes remains unclear; however, it is recommended that they be excluded from calculated aberration frequencies [1,37]. Unfortunately, in many studies, gaps are included in the total aberrations count, making their results incomparable with those of other studies [24,25], whereas some authors do not report gaps at all [22,50]. At INCT, we observe a very low frequency of gaps during aberration analysis. It appears that the frequency of gaps may be related to the quality of the preparations, as well as the specific chromosome staining methods used in individual laboratories.

### 2.2. Staining of Selected Chromosome Pairs Using FISH—Translocation Frequency Analysis

Fluorescence in situ hybridization (FISH) allows for the analysis of translocations—stable aberrations that are not visible with FPG staining. This method involves “painting” some of chromosomes (typically two or three pairs) by hybridizing their DNA with fluorescently labeled DNA probes (Figure 5) [1,51,52].

The FISH technique enables the analysis of all aberration types visible on Giemsa-stained (FPG) preparations, while additionally allowing for the detection of translocations that remain invisible with Giemsa staining. Translocations (Figure 5) do not cause structural problems during cell division; consequently, they are easily passed on to daughter cells, and their persistence in the cell population is significantly longer than that of unstable aberrations such as dicentric chromosomes.

Translocation analysis is more expensive than the analysis of FPG-stained preparations, as it involves additional costs for DNA probes, the use of a fluorescence microscope, and specialized analysis software. As a more precise method, translocation analysis is used to assess background (control) aberration frequencies and to study individuals occupationally exposed to radiation, heavy metals, or genotoxic environmental factors (e.g., [1,34,53,54]). Furthermore, the FISH method is employed to monitor aberration frequencies in nuclear power plant workers (e.g., [42,53,55]).

### 2.3. The M-FISH Technique—Staining of All Chromosomes

A more advanced, and simultaneously, the most expensive, version of the FISH method is M-FISH. Multicolor fluorescence in situ hybridization (M-FISH) allows for the analysis of translocations across the entire complement of chromosomes [52,56,57,58,59] (Figure 6). Each pair of homologous chromosomes is hybridized with a distinct set of DNA probes, making it possible to identify material originating from each individual pair of chromosomes.

The M-FISH analysis requires a fluorescence microscope equipped with a specific set of filters and dedicated computer software. Currently, two companies provide both M-FISH probe sets and aberration analysis software: MetaSystems (Altlussheim, Germany) and ASI (Applied Spectral Imaging, HiSKY, SKY, Carlsbad, CA, USA) [59,60]. While both systems are in use, MetaSystems is significantly more popular.

### 2.4. Description of Figure 1, Figure 2, Figure 3, Figure 4, Figure 5 and Figure 6

Micrographs presented in Figure 1, Figure 2, Figure 3, Figure 4, Figure 5 and Figure 6 are published only for illustrative purposes. All the photos were captured by employees of the Institute of Nuclear Chemistry and Technology in Warsaw between 2000 and 2023. All but one photo was captured in Warsaw at the Laboratory of Biological Dosimetry of INCT from the preparations used for the calibration curves for biological dosimetry. All donors provided informed consent for the use of their blood samples for the purpose of biological dosimetry laboratory experimentation and knowledge dissemination. Personal data were anonymized. The micrograph presented in Figure 6 was captured in Germany at GSI Helmholtzzentrum für Schwerionen-forschung GmbH Darmstad by an employee of INCT. The cells were purchased in form of a buffy coat. All photos represent human peripheral blood lymphocytes cultured, fixed and stained using the standard method [1,28,29].

## 3. Background (Control) Aberration Frequencies in Giemsa-Stained Preparations—A Literature Review

To determine the genotoxic effect of low-dose radiation, it is essential to compare the studied group (cohort) with a control donor database (background level of aberrations). Such databases are usually established by laboratories specializing in the cytogenetic effects of radiation, clinical laboratories dealing with genetic alterations associated with diseases (inherited genetic disorders or cancer), or by laboratories researching the genotoxic impact of occupational exposure such as medical radiation or environmental factors. These databases were initially developed through international scientific projects, and more recently, data from individual countries have been published (Table 1). The majority of available data is derived from the analysis of preparations stained using the FPG technique.

A control donor database should be sufficiently large and account for a range of factors, such as the donor’s age, sex, smoking habits, and geographic and ethnic diversity, among others. It appears that a sound strategy should involve the analysis of at least 500 cells from each donor, which is not always the case. Studies vary regarding the set of recognized aberrations; while dicentric chromosomes are analyzed by all researchers, tetraradial forms or gaps (which are not considered true aberrations) are described in only some publications. We would like to emphasize that Table 1 includes results from only a selection of published works. It was not our intention to analyze every study on this topic published to date, but rather to present a representative cross-section that justifies the development of a database for background (control) aberrations in Poland. Therefore, the publications included in Table 1 were selected from the published literature based on their relevance to the topic, historical coverage (1980s to the present), geographical diversity, and adequate reporting of chromosomal aberration data. The table is intended as an illustrative overview rather than a comprehensive review.

An analysis of the data in Table 1 leads to two primary conclusions: first, the frequency of dicentric chromosomes (potentially combined with centric rings) is very consistent across the studied populations, ranging from 0.00 to 3.26 per 1000 cells. Second, such consistency is lacking regarding other types of aberrations. For instance, the frequency of excess acentric fragments ranged from 0.4 to 22.0 per 1000 cells, and in three studies, this type of aberration was not analyzed at all. When considering the frequency of total aberrations, the range is very broad, spanning from 0.8 to as high as 45.7 aberrations per 1000 cells. It is notable that chromatid breaks and chromatid exchanges were analyzed in only 29 and 14 out of 42 papers, respectively, indicating that these types of aberrations are, in practice, less recognizable in human lymphocytes. Gaps were recorded only in 10 of 42 publications, but these are not aberrations, so this is understandable.

**Figure 7:**
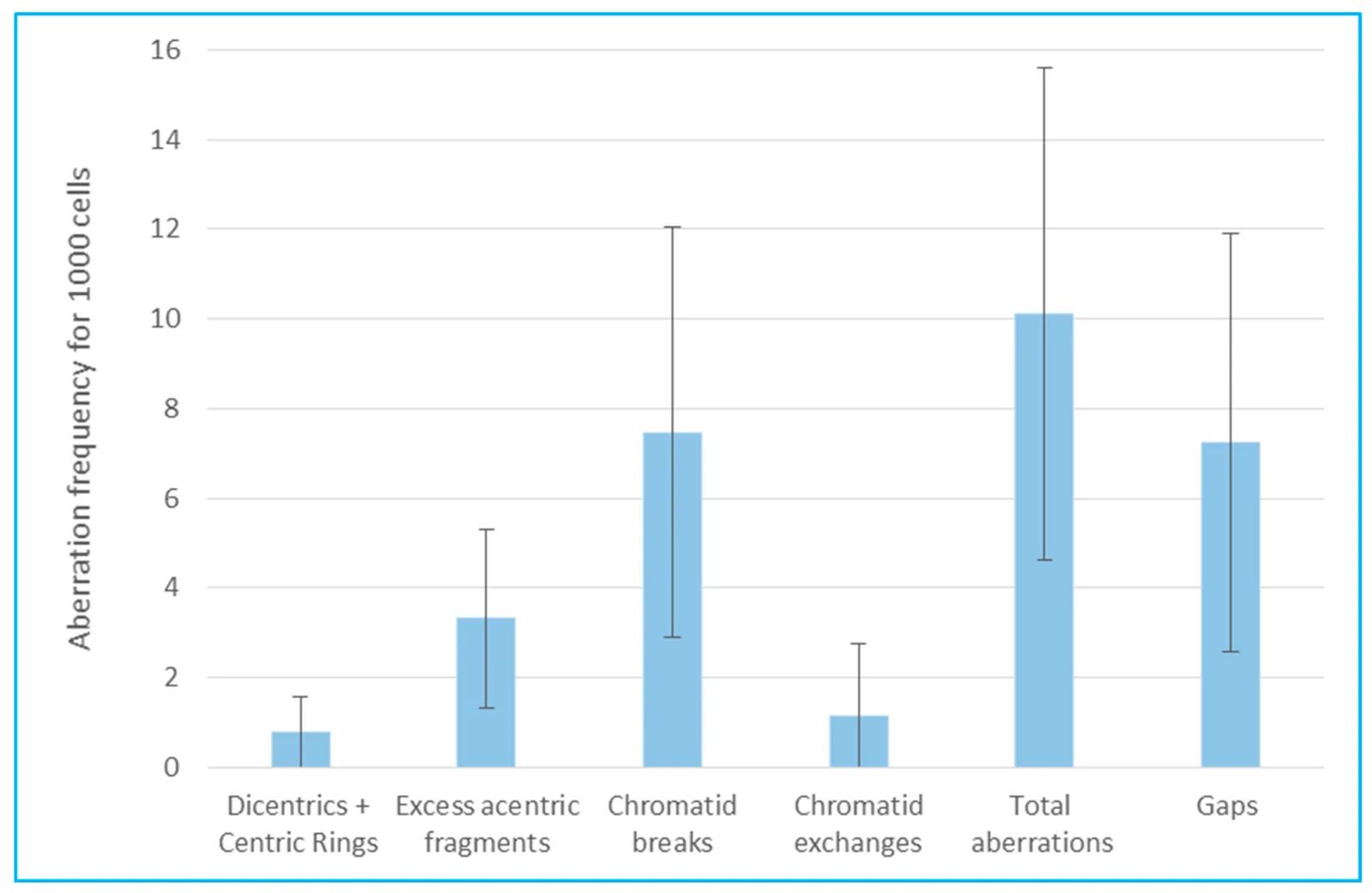
Mean frequencies of various types of aberrations, calculated as averages from the studies cited in Table 1, along with their standard deviations. Six papers are included in Table 1, with excessively low or high total aberration frequencies excluded from the data presented.

Figure 7 shows the mean frequencies of various types of aberrations, calculated as averages from the studies cited in Table 1, along with their standard deviations. It appears that an average total aberration frequency below 2.0 (found in 3 out of 42 cited studies) or above 25.0 per 1000 cells (found in 3 out of 42 cited studies) is unrealistic for control cells, as these figures deviate significantly from the data presented in the other works. The six previously mentioned studies with excessively low or high aberration frequencies were excluded from the data presented in Figure 7, and this is arbitrary decision. This decision was made to avoid the large standard deviation observed in Figure 7. Because the frequencies of various aberration types are well established, excessive deviation from these data typically indicates methodological differences between studies. The analysis of the graph confirms that dicentric chromosomes (+ centric rings) are the rarest type of aberration. The most frequently occurring aberrations are chromatid breaks. Chromatid discontinuities (gaps) are of high frequency as well.

## 4. Database of Background Chromosome Aberration Frequencies in Poland

It appears that a database of aberration levels in control donors in Poland was established only once, 25 years ago, in the Silesia region; however, the data presented in that publication grouped structural aberrations and “gaps” together, which prevents their comparison with results from other studies [25]. Data regarding aberration frequencies analyzed using the G-banding technique in control donors has also been published [82]. Again, while these results are highly valuable, as they demonstrate a statistically significant increase in aberration frequency with donor age, they cannot be compared with the data in Table 1. This is because the G-banding technique focuses on a different type of aberration, i.e., stable aberrations (translocations), which are not standardly analyzed in Giemsa-stained preparations. Given the development of nuclear power and the use of motorized microscopes coupled with high-resolution CCD cameras and dedicated aberration analysis software—providing superior analytical capabilities—it seems essential to establish a database of background aberration frequency values in Poland at this time.

A database of the background level of aberrations has been under development at the Institute of Nuclear Chemistry and Technology (INCT) in Warsaw for some time. Currently, it contains data from 13 healthy donors who, to our knowledge, have not been exposed to genotoxic factors. All donors resided in the vicinity of Warsaw (Masovian Voivodeship, Poland)—a lowland area characterized by low radon concentrations and an absence of major industrial pollution. The study group consisted of six men and seven women aged 21 to 68 years (mean age: 39.5 ± 15.6 years). Only one of the 13 participants smoked regularly (half a pack of cigarettes per day). All individuals underwent routine diagnostic imaging, including conventional radiography and CT scans; none had a history of nuclear medicine procedures or radiotherapy. All participants were office workers.

Blood samples from eight donors were collected between 2008 and 2012, at which time FPG-stained preparations were also produced. These slides were recently re-analyzed using a newer version of the MetaSystems software (MetaSystems, Metafer 4.3.14). Most of the preparations remained suitable for analysis after 15 years, despite being stored at room temperature. In cases where the staining quality had deteriorated, frozen samples were utilized, which were thawed and subsequently stained using the FPG technique. Samples from the remaining five donors were analyzed immediately after slide preparation in 2024 and 2025. The study is being conducted in accordance with the Declaration of Helsinki [83,84]. All donors provided informed consent for the use of their blood samples to study background aberration frequencies, and all donor personal data has been anonymized.

**Figure 8:**
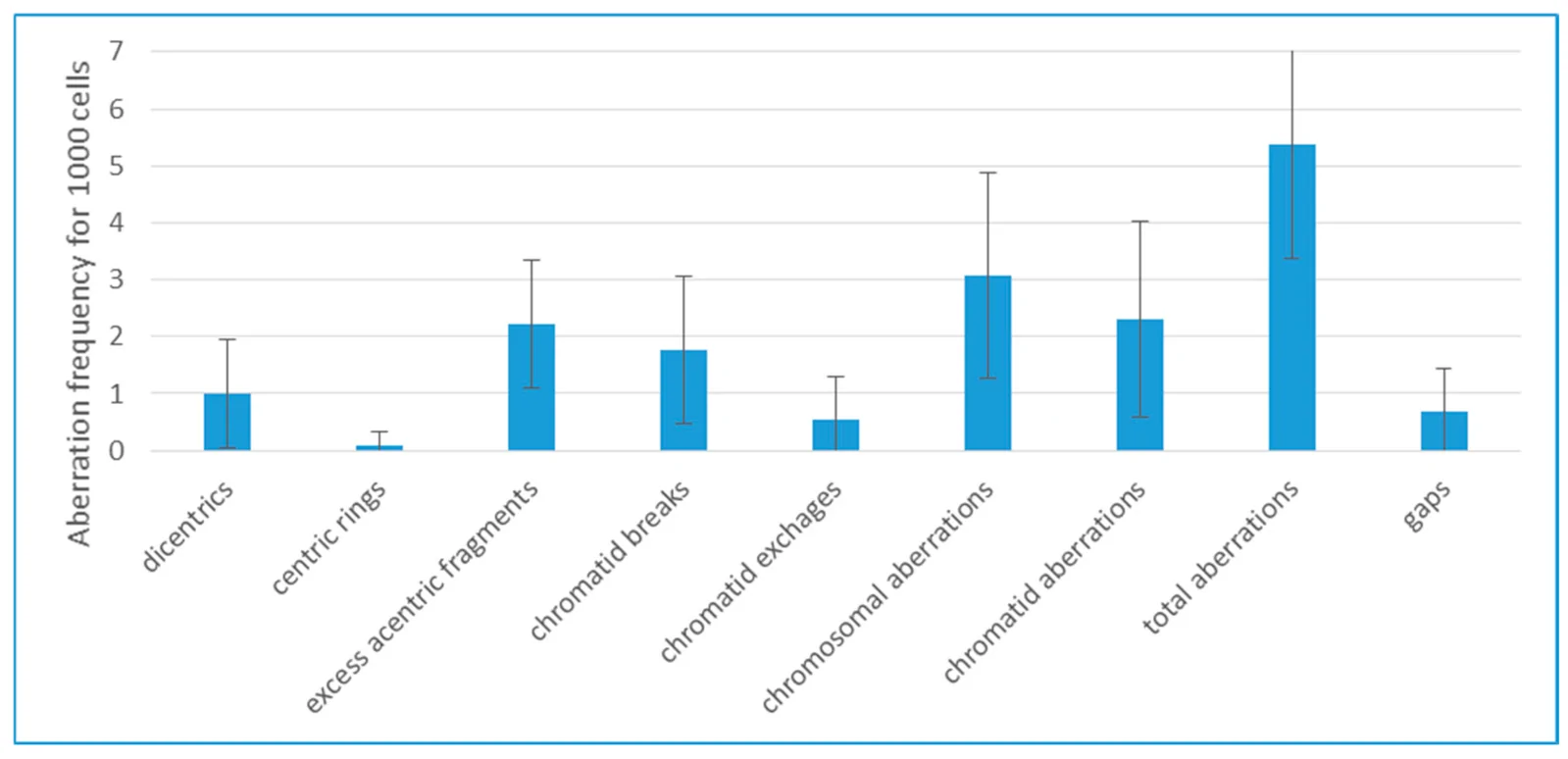
Mean frequencies of various types of aberrations, along with their standard deviations, obtained from the analysis of samples from the 13 control donors at the INCT in Poland.

Peripheral blood lymphocytes were stimulated to divide and were then cultured and fixed using the standard method [1,28,29]. The preparations were stained using the FPG technique and analyzed using a motorized Zeiss microscope coupled with Metasystems software. Approximately 1000 high-quality first-division metaphases were analyzed per donor. Slides from each donor were scored by two experienced cytogeneticists, each analyzing 500 cells. Aberrations were classified according to the IAEA manual. The mean aberration frequencies obtained from the analysis of samples from the 13 control donors are presented in Figure 8.

The results obtained fall within the range of data reported by other research groups, e.g., those presented in the Table 1. The frequency of dicentric chromosomes is consistent with the literature data, which typically cite values in the order of 0.5–1.0 dicentrics per 1000 cells [1] or about 1.0 dicentrics per 1000 cells (Table 1). Furthermore, the established distribution pattern between aberration types is maintained: centric rings are significantly less frequent than are dicentric chromosomes, while excess acentric fragments occur more frequently than do dicentrics [1,85]. The frequencies of chromatid breaks and gaps in the INCT data (Figure 8) are lower than the averages presented in Figure 7. It should be emphasized that all analyses were performed using a motorized Zeiss AxioImager.Z2 microscope and MetaSystems software (Metafer 4.3.14), which provides a level of mitotic image quality previously difficult to achieve, thereby ensuring high confidence in the analytical results. We hope that this database—though currently limited in size—will expand in the coming years and serve as a baseline for assessing the impact of the Polish nuclear power program on aberration frequencies among workers and residents living in the vicinity of nuclear facilities.

## 5. Risks of Cancer Development and the Frequency of Aberrations Among Nuclear Power Plant Workers

Nuclear power plant workers are exposed to various doses of ionizing radiation, as well as different types of radiation, such as gamma radiation, X-rays, beta radiation, and neutrons [36]. Generally, the doses absorbed by workers are recorded, and data for each individual can be found in systems such as the Korean National Dose Registry [55] or the National Registry for Radiation Workers in the United Kingdom [4]. Annual effective doses do not exceed 20 mSv (millisieverts); however, the highest recorded doses approach this limit [54]. Cumulative doses received over decades are sometimes measured in hundreds of millisieverts [5,6,7,8,55].

The research results regarding cancer incidence among nuclear industry workers remain inconclusive. A study published in 2007 by the International Agency for Research on Cancer (IARC) concerning cancer mortality among 400,000 nuclear industry workers across 15 countries showed increased cancer mortality in the low-dose range [5]. These studies demonstrated an increase in relative risk (RR) for mortality correlated with dose, both for all cancers combined and for leukemias (excluding lymphocytic leukemia), as well as lung cancers. The same results have been repeated by INWORKS (International Workers Study) program [6,7,8]. Similar findings have been obtained by numerous authors, such as Muirhead [4], who evaluated cancer incidence registries of occupationally exposed individuals in the United Kingdom, or Metz-Flamant et al. [9], who analyzed the incidence of solid tumors among nuclear industry workers in France.

However, other authors maintain that low doses of radiation in the nuclear industry do not lead to an increased risk of cancer induction or other diseases. Here are a few selected examples:

- A publication from Japan [86] indicating a similar cancer mortality rate between individuals exposed to low doses in the nuclear industry and the general Japanese population;
- A report by the Canadian Nuclear Safety Commission (2011) [87] regarding cancer mortality risk among nuclear industry workers in Canada, demonstrating no increase in risk;
- A publication from Germany [88] showing a decreased cancer mortality rate among nuclear power plant workers in Germany between 1991 and 1997;
- A series of review articles from Poland [89,90,91] generally demonstrating, based on the analysis of data from various publications, that radiation doses received in the nuclear power industry are not harmful.

There is a greater consensus that an increased frequency of chromosomal aberrations can be found among nuclear industry workers exposed to low, chronic doses of ionizing radiation. Such data, obtained through the analysis of Giemsa-stained preparations and chromosome painting (FISH), have been reported in numerous studies:

- Germany—a significantly increased frequency of dicentric chromosomes was found in 22 power plant workers with a mean cumulative dose of 390 mSv (dose range 270–530 mSv) [10];
- Finland—it was demonstrated that the frequency of translocations is higher among nuclear power plant workers who received an average cumulative dose of 100 mSv. Analysis of dicentric chromosome frequency did not reveal such a correlation [53,92];
- Bulgaria—aberration frequencies were studied using the FPG and FISH techniques; in both cases, significantly more aberrations were found in nuclear power plant workers exposed to low doses of radiation compared to the results for the controls [42,93];
- United Kingdom—a statistically significant correlation was shown between the external radiation dose and the frequency of translocations (three pairs of painted chromosomes) among nuclear power plant workers [11];
- Korea—the frequency of chromosomal and chromatid aberrations among nuclear power plant workers was significantly higher than in the control group and increased with the radiation dose [55].

To the best of our knowledge, no results have been published to date regarding the frequency of aberrations in the chromosomes of nuclear industry workers analyzed using the M-FISH technique, which appears to be the most accurate method.

An increased frequency of aberrations is a clear indicator of the body’s exposure to genotoxic factors—in the case of nuclear power plant workers, to ionizing radiation. On the other hand, many authors link an increased frequency of aberrations with the development or induction of the carcinogenesis process (for example, [12,49]). Thus, studying the level of aberrations induced by radiation doses accumulated over many years of work may, to some extent, help determine the risk of cancer development.

## 6. Conclusions

- The background frequency of aberrations in human lymphocytes is studied in many European countries as a fundamental element for monitoring potentially genotoxic factors, such as ionizing radiation.
- Three methods are used to monitor aberration frequency, i.e., most commonly, Giemsa staining (FPG), FISH, or M-FISH.
- There are issues regarding aberration type nomenclature in Giemsa-stained cells, especially regarding acentric fragments. Some standardization of nomenclature is necessary. It appears that the most commonly used terms for such types of aberrations are acentrics, acentric fragments, or excess acentric fragments, and one of these names should be consistently selected.
- In the context of nuclear power development, there is a need to monitor the impact of low, chronic radiation doses, accumulated over many years, on the level of cytogenetic changes.
- Until now, a database compiling the background level of aberrations in donors in Poland has been lacking. The Laboratory of Dosimetry and Radiation Protection at the Institute of Nuclear Chemistry and Technology in Warsaw—equipped with modern image analysis systems integrated with microscopes (Metasystems), appropriate procedures, and competent personnel—is the ideal facility to establish a database of background frequency aberration levels in healthy donors. So far, samples from 13 donors have been analyzed, comprising 13,000 cells. We hope that this database—though currently limited in size—will expand in the coming years and serve as a baseline for assessing the impact of the Polish nuclear power program on aberration frequencies among workers and residents living in the vicinity of nuclear facilities.

## Figures and Tables

**Table 1:** Exemplary data of frequencies of a background set of aberrations in Giemsa (FPG)-stained preparations.

Publication	Country	Number of Control Donors	Number of Analyzed Cells	Dic * (Dic + Centric Rings)/1000 Cells	Excess Acentric Fragments/1000 Cells	Chromatid Breaks/1000 Cells	Chromatid Exchanges/1000 Cells	Total Aberrations/1000 Cells	Gaps/ 1000 Cells
Barcinski et al., (1975) [13]	Brazil	147	9001	(0.67)	8.6	n.r.	36.4	45.7	n.r.
Lloyd et al., (1980) [61]	Review	2000	211,661	0.78	3.7	n.r.	n.r.	n.r.	n.r.
Gundy et al., (1983) [62]	Hungary	175	17,500	(0.69)	4.6	6.4	n.r.	11.7	n.r.
Bender et al., (1988) [33]	Review	2830	303,233	1.42 (1.65)	2.81	n.r.	n.r.	n.r.	n.r.
Bauchinger et al., (1994) [14]	Germany	85	45,952	(0.52)	n.r.	n.r.	n.r.	n.r.	n.r.
Carbonell et. al., (1996) [35]	Spain	85	8194	0.80	10.3	27.6	1.4	44.6	n.r.
Lazutka et al., (1999) [26]	Lithuania	50	8420	(3.26)	7.4	10.7	1.2	21.7	n.r.
Stephan et al., (1999) [63]	Germany	53	54,689	1.15 (1.23)	2.6	7.0	0.4	11.2	n.r.
Heimers, A., (2000) [64]	Germany	10	10,065	0.30	2.6	n.r	n.r	2.9	n.r.
Testa et al., (2001) [23]	Kazakhstan	20	4000	(0.75)	5.5	n.r.	n.r.	6.3	n.r.
Lalic et al. (2002) [65]	Croatia	14	2800	0.00	6.1	2.0	n.r	8.0	5.8
Svyatova et al., (2002) [66]	Kazakhstan	25	8697	(0.46)	2.0	6.5	3.4	12.4	n.r.
Umadevi et al., (2003) [20]	India	138	20,700	3.09	0.4	16.1	n.r	19.7	13.6
Abil’dinova et al., (2003) [67]	Kazakhstan	25	8716	(0.23)	2.0	5.6	3.7	11.5	n.r.
Lalic et al., (2004) [68]	Croatia	12	2400	0.00	0.4	0.4	n.r	0.8	7.5
Maffei et al., (2004) [69]	Italy	35	17,500	0.17	2.6	7.7	n.r	10.8	5.1
Atanasova et al., (2004) [34]	Bulgaria	9	4200	0.00	3.2	7.2	0.0	10.8	n.r.
M. Bilban et al., (2005) [70]	Slovenia	153	30,600	(0.00)	2.0	15.0	n.r	16.0	n.r.
Tanaka et al., (2006) [71]	Kazakhstan	46	14,192	(0.78)	n.r.	14.4	n.r	n.r	n.r.
Takeichi et al., (2006) [72]	Kazakhstan	18	6600	(0.15)	1.1	n.r.	n.r	1.3	n.r.
Kasuba et al., (2008) [17]	Croatia	200	40,000	(0.55)	2.1	n.r.	0.0	2.7	n.r.
Milić et al., (2008) [21]	Croatia	40	8000	0.10	3.4	3.5	0.0	7.0	n.r.
Zakeri et al., (2010) [73]	Iran	35	7000	0.43	1.6	3.6	n.r	12.8	7.7
Zakeri et al., (2010) [74]	Iran	37	22,500	(0.40)	4.2	9.6	n.r	12.7	8.5
Hristova et al., (2013) [42]	Bulgaria	45	21,853	(0.30)	9.2	4.4	0.6	14.5	n.r
Jovicic et al., (2013) [27]	Serbia	32	6400	n.r	2.3	1.2	0.0	3.5	n.r.
Sakly et al., (2013) [75]	Tunisia	27	2700	(0.56)	3.6	n.r	n.r	4.2	0.2
Nefic and Musanovic, (2014) [39]	Bosnia and Herzegovina	100	10,000	5.20	14.0	9.2	n.r.	28.4	n.r.
Santovito et al., (2014) [18]	Italy	21	4200	0.95	3.6	7.6	0.0	12.1	n.r.
Santovito et al., (2016) [40]	Italy	101	20,200	0.54 (1.93)	5.5	10.3	n.r.	17.3	13.5
Pajic et al., (2016) [76]	Serbia	40	8000	0.63	2.8	1.6	n.r	5.0	n.r.
Kumar et al., (2017) [77]	India	5	4525	0.88	4.0	3.8	n.r	8.6	n.r.
Karuppasamy et al., (2018) [15]	India	97	25,359	(1.54)	6.7	n.r.	4.1	8.2	n.r.
Fang et al., (2019) [19]	China	159	15,900	(0.31)	1.1	n.r	n.r	1.3	n.r.
Djansugurova et al., (2020) [78]	Kazakhstan	236	22,632	(0.13)	n.r.	n.r.	n.r.	11.7	n.r.
Shafie et al. (2020) [79]	Iran	35	17,500	(0.51)	1.8	n.r	n.r	2.3	3.4
Farkas et al., (2021) [38]	Hungary	1050	105,000	(1.20)	1.6	14.3	n.r.	18.4	n.r
Kenzhina et al., (2022) [22]	Kazakhstan	40	168,362	(0.79)	1.3	n.r	n.r	2.1	n.r.
Kim et al., (2022) [55]	South Korea	59	29,500	0.28	0.8	7.2	0.2	8.4	n.r.
Gulati et al., (2024) [80]	Germany	12	12,000	(5.0)	22.0	n.r	n.r	27.0	n.r.
Farkas et al., (2025) [50]	Hungary	732	73,200	(1.30)	2.8	12.5	n.r	16.5	n.r.
Kostova et al., (2026) [81]	Bulgaria	9	1800	(0.10)	1.7	1.1	n.r	2.8	n.r.

* Dic—dicentric chromosomes; n.r.—data not recorded.

## Data Availability

The raw data supporting the conclusions of this article will be made available by the authors on request.

## Abbreviations

The following abbreviations are used in this manuscript:

DAPI	4′,6-diamidino-2-phenylindole
FISH	Fluorescence In Situ Hybridization
FPG	Fluorescent Plus Giemsa staining
IAEA	International Atomic Energy Agency
INCT	Institute of Nuclear Chemistry and Technology
ISCN	International System for Human Cytogenomic Nomenclature
M-FISH	Multicolor Fluorescence In Situ Hybridization
mSv	millisievert
